# Speckle-tracking echocardiography provides sensitive measurements of subtle early alterations associated with cardiac dysfunction in T2DM rats

**DOI:** 10.1186/s12872-023-03239-2

**Published:** 2023-05-22

**Authors:** Yanchao Qi, Zhiyan Chen, Bingyan Guo, Zhe Liu, Lijie Wang, Suyun Liu, Lixiang Xue, Meifang Ma, Yajuan Yin, Yongjun Li, Gang Liu

**Affiliations:** 1 Department of Cardiology, The First Hospital of Hebei Medical University, Shijiazhuang, 050031 Hebei People’s Republic of China; 2 Heart Center, The First Hospital of Hebei Medical University, Shijiazhuang, 050031 Hebei People’s Republic of China; 3 Department of Cardiology, The Second Hospital of Hebei Medical University, Shijiazhuang, 050000 Hebei People’s Republic of China; 4 Center of Basic Medical Research, Peking University Third Hospital, Beijing, 100191 People’s Republic of China; 5 Department of Cardiology, Handan Central Hospital, Handan, 056008 Hebei People’s Republic of China; 6 Hebei International Joint Research Center for Structural Heart Disease, Shijiazhuang, 050031 Hebei People’s Republic of China; 7 Hebei Key Laboratory of Cardiac Injury Repair Mechanism Study, Shijiazhuang, 050031 Hebei People’s Republic of China; 8 Hebei Key Laboratory of Heart and Metabolism, Shijiazhuang, 050031 People’s Republic of China

**Keywords:** Diabetic cardiomyopathy, Myocardial damage, RhoA, ROCK signalling pathway inhibition, Cardiac function, Speckle-tracking echocardiography

## Abstract

**Background:**

Diabetic cardiomyopathy results in cardiac structural and functional abnormalities. Previous studies have demonstrated that inhibiting the RhoA/ROCK signalling pathway increases the injury resistance of cardiomyocytes. The early detection of cardiac structural and functional alterations may facilitate an improved understanding of the pathophysiologic progress and guide therapy. This study aimed to identify the optimal diagnostic measures for the subtle early alterations of cardiac dysfunction in type 2 diabetes mellitus (T2DM) rats.

**Methods:**

Twenty-four rat models were divided into four groups and received treatments for 4 weeks: the CON group (control rats), the DM group (T2DM rats), the DMF group (T2DM rats receiving fasudil) and the CONF group (control rats receiving fasudil) group. Left ventricular (LV) structure was quantified by histological staining and transmission electron microscopy. LV function and myocardial deformation were assessed by high-frequency echocardiography.

**Results:**

Treatment with fasudil, a ROCK inhibitor, significantly protected against diabetes-induced myocardial hypertrophy, fibrosis and mitochondrial dysfunction. Impaired LV performance was found in T2DM rats, as evidenced by significant reductions in the ejection fraction (EF), fractional shortening (FS) and the mitral valve (MV) E/A ratio (which decreased 26%, 34% and 20%, respectively). Fasudil failed to improve the conventional ultrasonic parameters in T2DM rats, but the myocardial deformation measured by speckle-tracking echocardiography (STE) were significantly improved (global circumferential strain, GCS: *P* = 0.003; GCS rate, GCSR: *P* = 0.021). When receiver operating characteristic (ROC) curves were used in combination with linear regression analysis, STE parameters were found to be characterized by both optimal prediction of cardiac damage [AUC (95% CI): fractional area change, FAC: 0.927 (0.744, 0.993); GCS: 0.819 (0.610, 0.945); GCSR: 0.899 (0.707, 0.984)] and stronger correlations with cardiac fibrosis (FAC: r = -0.825; GCS: r = 0.772; GCSR: r = 0.829) than conventional parameters.

**Conclusion:**

The results suggest that STE parameters are more sensitive and specific than conventional parameters in predicting the subtle cardiac functional changes that occur in the early stage, providing new insight into the management of diabetic cardiomyopathy.

**Supplementary Information:**

The online version contains supplementary material available at 10.1186/s12872-023-03239-2.

## Background

Diabetes mellitus (DM) has a high global prevalence, with more than 450 million adult patients worldwide, and the number is expected to reach 693 million by 2045 [1]. Diabetic cardiomyopathy, a serious cardiovascular complication that is particularly common in type 2 diabetes mellitus (T2DM) [2], contributes to the incidence of the composite endpoint of worsening of heart failure (HF) or death. The term “diabetic cardiomyopathy” is defined as a series of structural abnormalities, including diffuse myocardial fibrosis; cardiomyocyte hypertrophy; coronary microvascular disorder; and cardiac dysfunction independent of cardiovascular risk factors, such as hypertension, atherosclerosis or coronary artery disease [3, 4].

As the underlying mechanisms have increasingly come to light, the RhoA/ROCK signalling pathway is proposed to contribute to the pathogenesis of diabetic cardiomyopathy [5–7]. Rho-associated coiled-coil containing kinases (ROCKs) belong to the AGC serine/threonine kinase family and are downstream effectors of RhoA, a member of the small GTP-binding protein family [8]. Activated ROCK contributes to cardiomyocyte apoptosis, cardiac hypertrophy and fibrosis [5, 9, 10], and it also mediates vascular smooth muscle cell contraction and the production of several proinflammatory, thrombogenic and fibrogenic molecules [5]. Our previous studies have shown that inhibiting the RhoA/ROCK signalling pathway improved structural abnormalities in diabetic hearts [11–13]. Changes in cardiac structure are usually accompanied by functional alterations. Early assessment of cardiac performance during therapy may facilitate a better understanding of the pathophysiologic progression of diabetic cardiomyopathy and guide its treatment.

Transthoracic echocardiography is a standard noninvasive method of cardiac function assessment in both animal models and humans. Conventional parameters, such as ejection fraction (EF) and fractional shortening (FS), are classical and widely used markers of cardiac function [14]. However, their modest sensitivity and technical limitations make them unable to analyse the subtle changes associated with myocardial damage [15]. Speckle-tracking echocardiography (STE) is a highly sensitive technique that characterizes myocardial deformation by global and segmental strain [16–19]. Parameters derived from STE are valuable for describing the left ventricular (LV) dynamic changes in active relaxation and contractility in type 1 diabetes mellitus (T1DM) and T2DM animal models [20]. Moreover, previous studies have suggested that myocardial strain and strain rate can detect subtle differences in pressure-overload-induced myocardial remodelling between juvenile and adult rats [16]. Most notably, longitudinal strain (LS) parameters are well-accepted surrogates of subendocardial fibrosis and may serve as diagnostic markers of microstructural remodelling [17]. However, evidence is lacking as to whether STE can be used as a sensitive diagnostic method for diabetic cardiomyopathy to evaluate subtle changes in cardiac function and predict the early effects of treatment.

In the present study, we aimed to investigate the subtle changes in cardiac structure and function in T2DM rats after treatment with a Rho-kinase inhibitor. By combining comprehensive STE analyses with several other measures, we sought to provide translational evidence for diabetic cardiomyopathy management. We tested the hypothesis that the early protective actions of inhibiting the RhoA/ROCK signalling pathway on cardiac remodelling in T2DM rats are detectable by assessing subtle cardiac functional alterations using STE.

## Methods

### Animals

The experimental protocols used in this study followed the Guide for the Care and Use of Laboratory Animals published by the US National Institutes of Health (1996) and were approved by the research ethics committee of Hebei Medical University. Four-week-old Wistar rats (*Rattus norvegicus*) were obtained from the Vital River Laboratory (Beijing, China). All rats were housed in a temperature-controlled room on a 12/12 h light/dark cycle with free access to water and standard rodent chow.

Following 1 week of acclimatization, the rats were randomly divided into two groups: control and diabetes. After being fed a high-fat diet (HFD, 60 kcal% fat, D12492, Vital River Laboratory, Beijing, China) for 6 weeks, the rats were fasted for 8 h and then intraperitoneally injected with a single dose of streptozotocin (STZ, 35 mg/kg; S0130, Sigma, USA) dissolved in 0.1 M citrate buffer (pH 4.5) to induce T2DM as previously described [21]. Control rats received a single intraperitoneal injection of citrate buffer alone and were fed the same normal diet as before. At 3 days, 1 week and 4 weeks of STZ injection, the fasting blood glucose (FBG) level was measured with a blood glucose meter (yuwell580, China) using tail vein blood samples. FBG > 11.1 mmol/l was considered to indicate diabetes. All diabetic rats continued to receive the HFD for 24 weeks.

### Experimental design

Twenty-four weeks after the diabetes model was initially generated, the normal and diabetic rats were randomly subdivided into 2 groups per condition (at least 6 rats in each group), resulting in 4 groups: the CON group (control rats), the DM group (T2DM rats), the DMF group (T2DM rats + receiving fasudil) and the CONF group (control rats receiving fasudil). The rats from the DMF group and the CONF group were treated with fasudil (10 mg/kg/d; Hongri, Tianjin, China) by intraperitoneal injection. The CON group and DM group received an equal volume of saline intraperitoneally each day. After 4 weeks of treatment, rats were sacrificed so that heart tissues could be collected for further analysis (Fig. S1).

### Histological analysis

LV samples from all rats were harvested and fixed with 4% paraformaldehyde for 8 h at room temperature and then embedded in paraffin. Subsequently, the heart samples were cut into 5-μm-thick sections and separately stained with haematoxylin–eosin (HE) and Masson’s trichrome as previously described [22]. Digital images were obtained by light microscopy (BX51T-PHD-J11, OLYMPUS, Tokyo, Japan). Image analysis was performed by using Image-Pro Plus 6.0 software (Media Cybernetics, USA) to quantify the morphological changes and collagen content of the heart tissues. Data on cardiomyocyte cross-sectional area (CSA) were collected from 30 representative cardiomyocytes per field in 3 random fields per slide. The volume fraction of collagen (CVF) was calculated by the formula CVF % = average collagen area/area of total field × 100%.

### Transmission Electron Microscopy (TEM)

TEM was performed to examine the cardiac ultrastructure according to a previous protocol [23, 24]. LV samples were harvested and fixed in 2.5% glutaraldehyde overnight at 4 °C and postfixed in 1% osmic acid. After dehydration, the tissues were embedded with Epon 812 and baked at 60 °C for 36 h. Ultrathin Sects. (50 ~ 60 nm) were obtained and stained with 3% uranyl acetate–lead citrate and then examined with a TEM (H-7650, HITACHI, Japan). The mitochondrial morphological parameters were calculated in six random fields per slide, and the numbers from six slides were averaged for each rat. The following mitochondrial morphological characteristics were quantified by Image-Pro Plus 6.0 software (Media Cybernetics, USA): (1) mitochondrial area; (2) aspect ratio: defined as the ratio between the major and minor axis lengths of a mitochondrion; (3) degree of branching: defined as (Pm^2^)/(4pAm), where Pm is the perimeter of a mitochondrion and Am is the area; and (4) number of vacuolized mitochondria.

### Echocardiography

Echocardiography was used to evaluate cardiac function in all of the rats after 4 weeks of drug administration but before sacrifice, as previously described [25]. Ultrasound imaging was obtained with a 4 A VisualSonics high-resolution Vevo 2100 system (VisualSonics Inc., Toronto, Canada) with a 16-MHz linear transducer (MS-250). In brief, rats were anaesthetized with 2% isoflurane and placed in the supine position on a heated platform (to maintain a body temperature of 37 °C) for continuous electrocardiographic monitoring. After the heart rate (HR) was stable, B-mode images were acquired in the parasternal long-axis and short-axis views, and M-mode images were acquired in the parasternal short-axis view at the mid-papillary level. Then, an apical four-chamber view was acquired to measure the mitral valve flow via a pulsed-wave (PW) Doppler method. All the parameters were assessed during at least 3 consecutive cardiac cycles, and the mean was calculated. All analyses were performed in a blinded manner.

### Conventional echocardiography

The conventional echocardiographic parameters, including the left ventricular end-diastolic inner dimension (LVIDd), left ventricular end-systolic inner dimension (LVIDs), diastolic left ventricular anterior wall thickness (LVAWd), systolic left ventricular anterior wall thickness (LVAWs), diastolic left ventricular posterior wall thickness (LVPWd), systolic left ventricular posterior wall thickness (LVPWs), left ventricular end-diastolic volume (LVEDV), left ventricular end-systolic volume (LVESV), EF, FS, stroke volume (SV) and cardiac output (CO), were obtained and calculated from the M-mode images captured in the parasternal short-axis view to assess systolic function. The early (E) and late (A) peak mitral valve flow velocities were analysed using PW Doppler imaging in the apical four-chamber view to evaluate diastolic function.

### STE

STE was obtained in the parasternal long-axis and short-axis views [18, 26]. B-mode images with a frame rate above 200 frames/s were used, and the LV endocardium and epicardium were then traced manually in end diastole. For the long-axis view, the global longitudinal strain (GLS) and strain rate (GLSR) were calculated, and for the short-axis view, the global radial strain (GRS), global radial strain rate (GRSR), global circumferential strain (GCS) and global circumferential strain rate (GCSR) were obtained. LV fractional area change (FAC) was calculated by the formula FAC = (end diastolic area – end collecting area)/end diastolic area × 100%. All the parameters were averaged across the 6 segments of the LV.

### Quantitative Real-Time PCR (qRT‒PCR)

Total mRNA was extracted from the LV samples using TRIzol reagent (Invitrogen, Carlsbad, CA) according to the manufacturer’s protocol [27]. The detailed methodology is available in the online-only Data Supplement.

### Statistical analysis

The values are presented as the means ± SDs. Differences among four groups were analysed using one-way ANOVA. Receiver operating characteristic (ROC) curves were studied to determine the sensitivity and specificity of cardiac functional parameters in detecting myocardial damage and the optimal cutoff value for diagnosis. The areas under the ROC curves (AUC) and the comparison between the AUC of 2 ROC curves were calculated using the MedCalc ﻿v 19.0.7 software (MedCalc Software, Mariakerke, Belgium). Linear regression analysis was performed between continuous variables using Pearson’s correlation coefficient (r). Statistical analysis was performed with GraphPad Prism software 8.0 (GraphPad Software Inc., USA) and IBM SPSS Statistics 26.0 (IBM Corporation, USA). *P* values < 0.05 were considered statistically significant.

## Results

### ROCK inhibition improved diabetes-induced cardiac dysfunction in both microstructure and mitochondria dynamics

**Fig. 1:**
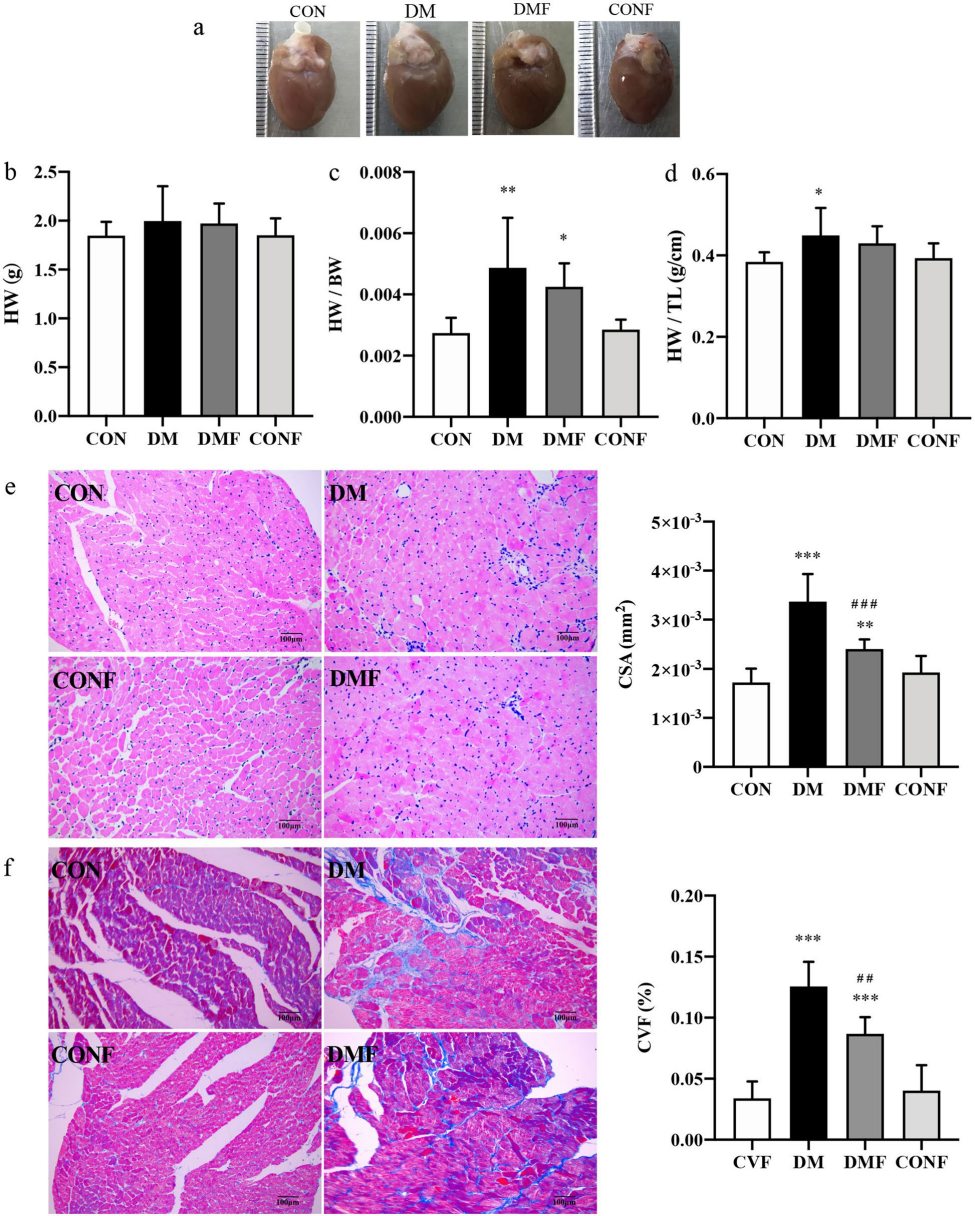
Changes in cardiac remodelling in rats. **a** Heart tissue specimens from the rats are shown, and **b** HW, **c** HW/BW and **d** HW/TL were calculated. **e** Representative images of cardiac cross sections stained with HE were obtained (magnification 200 ×), and the CSA of cardiomyocytes was calculated. Scale bar: 100 µm. **f** Representative histology of mid-myocardial cross-sections performed with Masson’s trichrome staining was acquired (magnification 200 ×). Scale bar: 100 μm. Quantification of collagen content was analysed. HW: heart weight, HW/BW: heart weight / body weight, HW/TL: heart weight / tibia length, CSA: cross sectional area, CVF: collagen volume fraction. Values are presented as the means ± SDs. *P < 0.05, **P < 0.01, ***P < 0.001 *vs* CON group by one-way ANOVA; ^#^*P* < 0.05, ^##^*P* < 0.01, ^###^*P* < 0.001 *vs* DM group by one-way ANOVA

We established a T2DM rat model stably characterized by a 2.8-fold increase in FBG and an approximately 35% decrease in ﻿body weight (BW) compared with normal rats (Table 1). However, short-term ROCK inhibition by fasudil had no effect on BW or FBG (Table 1). To observe the cardiac structural ﻿changes induced by diabetes (Fig. 1a), we performed HE staining and Masson’s trichrome staining. The heart weight (HW) was slightly increased in the DM group (Fig. 1b). However, significantly increased heart weight/body weight (HW/BW) and heart weight/tibia length (HW/TL) ratios were observed (*P* < 0.05, Fig. 1c and d) in the T2DM rats, as well as obvious cardiomyocyte hypertrophy reflected by a 1.9-fold increase in the CSA of myocardial cells (*P* < 0.001, Fig. 1e). Irregular fibre arrangement, broken nuclear membranes and ﻿inflammatory cell infiltration were also ﻿found in the DM group, whose CVF was increased by 3.7-fold. Fasudil alleviated pathological injury in T2DM rats (*P* < 0.001, Fig. 1f) but did not completely normalize the cardiac tissue: the treated rats still showed disorganized myocardial fibres and serious cardiomyocyte hypertrophy.

**Table 1:** Basic characteristics of the rats

	CON	DM	DMF	CONF
FBG (mmol/l)	6.07 ± 0.48	16.95 ± 1.76^***^	17.84 ± 3.20^***^	5.78 ± 0.55
BW (g)	689 ± 82	446 ± 151^***^	469 ± 52^***^	656 ± 37
HW (g)	1.85 ± 0.14	2.00 ± 0.36	1.97 ± 0.20	1.85 ± 0.17
LVW (g)	1.32 ± 0.19	1.44 ± 0.28	1.42 ± 0.16	1.31 ± 0.14
TL (cm)	4.81 ± 0.09	4.44 ± 0.17^***^	4.59 ± 0.08^**#^	4.71 ± 0.04
HW/BW	0.0027 ± 0.0005	0.0049 ± 0.0016^**^	0.0043 ± 0.0008^*^	0.0029 ± 0.0003
HW/TL	0.38 ± 0.02	0.45 ± 0.07^*^	0.43 ± 0.04	0.39 ± 0.04
LVW/HW	0.71 ± 0.06	0.72 ± 0.02	0.72 ± 0.04	0.71 ± 0.02

*FBG* fasting blood glucose, *BW* body weight, *HW* heart weight, *LVW* left ventricle weight, *TL* tibia length, *HW/BW* heart weight / body weight, *HW/TL* heart weight / tibia length, *LVW/HW* left ventricle weight / heart weight. Values are presented as the means ± SDs

^*^P < 0.05

^**^P < 0.01

^***^P < 0.001 *vs* CON group by one-way ANOVA

^#^*P* < 0.05

^##^*P* < 0.01

^###^*P* < 0.001 *vs* DM group by one-way ANOVA

**Fig. 2:**
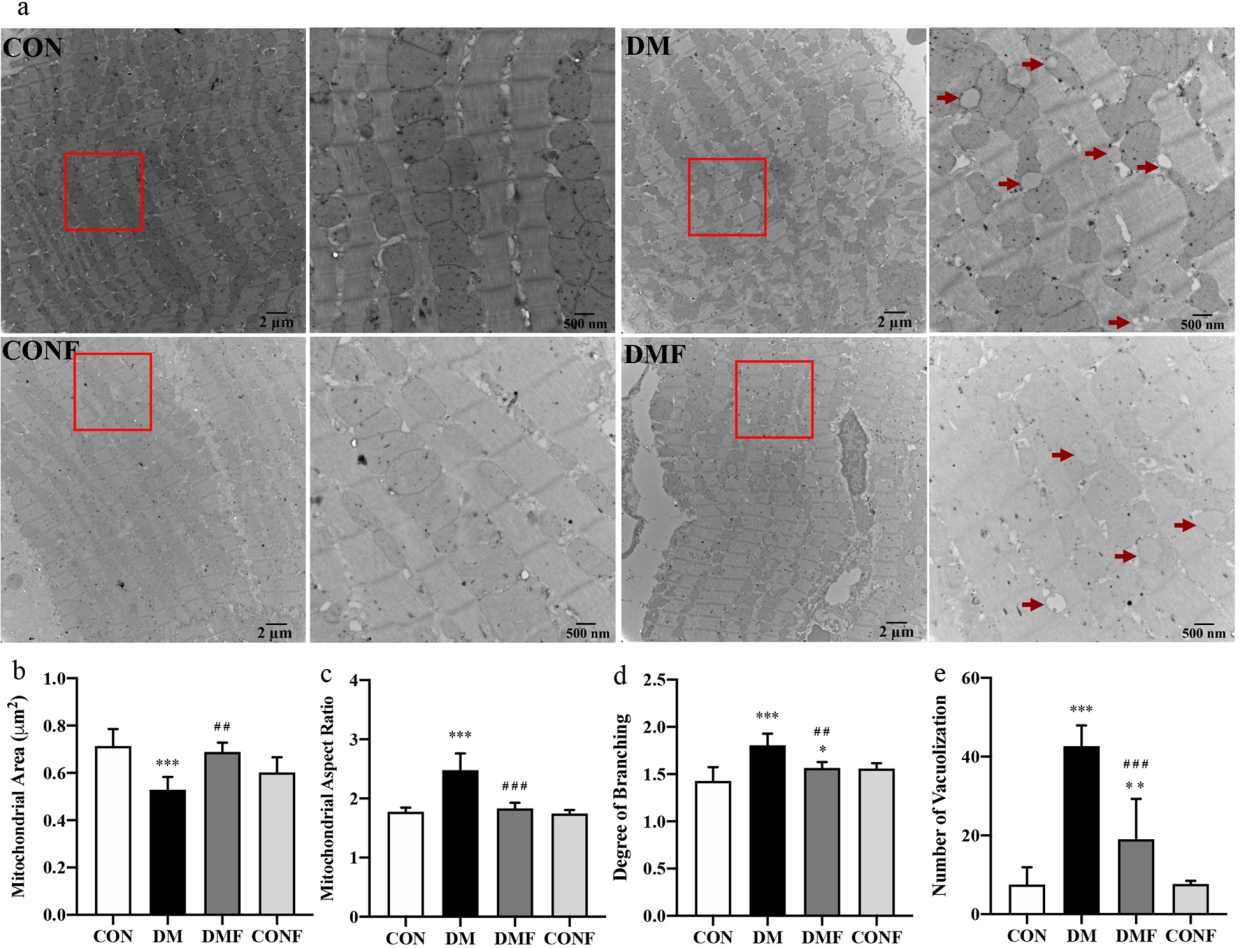
The cardiac ultrastructure was measured by TEM. **a** Representative images of the hearts obtained from TEM are shown. Scale bars: 2 µm and 500 nm. Arrows indicate mitochondria with vacuolization. **b** Mitochondrial area, **c** aspect ratio, **d** degree of branching and **e** number of mitochondria with vacuolization were analysed. ﻿Values are presented as the means ± SDs. *P < 0.05, **P < 0.01, ***P < 0.001 *vs* CON group by one-way ANOVA; ^#^*P* < 0.05, ^##^*P* < 0.01, ^###^*P* < 0.001 *vs* DM group by one-way ANOVA

Mitochondria provide energy for cardiomyocytes, serving as the power source for cardiac function. To further confirm the cardioprotective effect of ROCK inhibition, the mitochondrial morphology characteristics were determined by TEM, directly revealing ultrastructure changes in cardiomyocytes. Remarkable changes in mitochondrial morphology were noted in the T2DM rats, including a smaller mitochondrial area, an increased aspect ratio and degree of mitochondrial branching, and an increased number of distorted and vacuolated mitochondria (Fig. 2a), which indicated progressive impairments in mitochondrial function. Short-term treatment with fasudil significantly restored the mitochondrial area (a 1.2-fold increase,* P* = 0.005, Fig. 2b), reduced the mitochondrial aspect ratio (a 26% decrease, *P* < 0.001, Fig. 2c), reduced the degree of mitochondrial branching (a 13% decrease, *P* = 0.001, Fig. 2d) and alleviated vacuolization in mitochondria (a 55% decrease, *P* < 0.001, Fig. 2e) in the DMF group compared with the DM group. In addition, fasudil improved the imbalance of mitochondrial division and fusion by upregulating Mfn1 and Mfn2 mRNA transcription and downregulating Drp1 and Fis1 mRNA transcription (Fig. S2). These results confirmed the beneficial effects of fasudil on improving diabetes-induced mitochondrial dysfunction by maintaining mitochondrial dynamics stability.

### Conventional echocardiography was insensitive to subtle functional improvements in T2DM rats

**Fig. 3:**
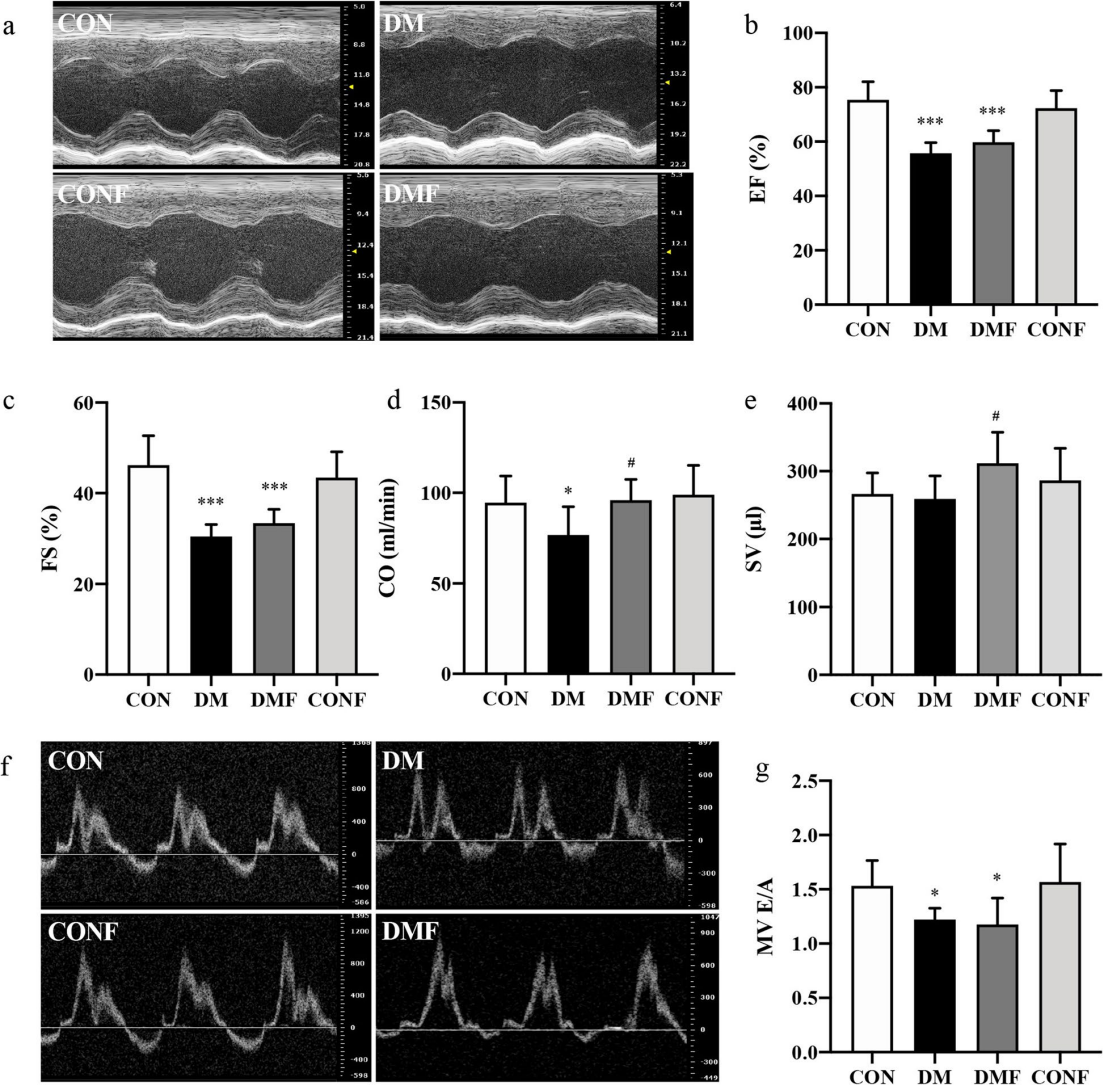
Cardiac dysfunction measured by conventional echocardiography. **a** The M-mode images in the parasternal short axis of the rats were acquired, and **b** EF, **c** FS, **d** CO and **e** SV were calculated. **f** The pulsed wave Doppler of the mitral valve flow was obtained, and **g** the peak velocity blood flow in early diastole (the E wave) to peak velocity flow in late diastole (the A wave) ratio was analysed. EF: ejection fraction, FS: fractional shortening, CO: cardiac output, SV: stroke volume, MV E/A: mitral valve E/A. Values are presented as the means ± SDs. *P < 0.05, **P < 0.01, ***P < 0.001 *vs* CON group by one-way ANOVA; ^#^*P* < 0.05, ^##^*P* < 0.01, ^###^*P* < 0.001 *vs* DM group by one-way ANOVA

The T2DM rats showed a noteworthy decline in HR, from 354 to 295 bmp, and an enlargement in LV characterized by increased LVIDd and LVEDV compared with the CON group (Table S2). However, fasudil had no significant effect on the HR in the T2DM rats. The T2DM rats had impaired LV systolic performance, as evidenced by reductions in EF and FS by 26% and 34%, respectively (Fig. 3a, b, c and Table S2). Meanwhile, CO was significantly depressed in the DM group, and CO and SV were increased in the DMF group (*P* < 0.05, Fig. 3d&e), but EF and FS did not show the same trend (Fig. 3b and c). In addition, cardiac diastolic dysfunction was evidenced by a 20% reduction in mitral valve (MV) E/A in the DM group compared to the controls (*P* < 0.05, Fig. 3f and g). Interestingly, a 4-week treatment with fasudil failed to improve the mitral valve E/A in the T2DM rats (*P* > 0.05, Fig. 3g). Cardiac function measured by conventional echocardiography showed almost no change with the ROCK inhibitor. This indicated that the conventional ultrasound parameters were insensitive to subtle changes in cardiac function when the tissue structure was improved slightly by fasudil.

### Alterations in myocardial deformation were observed in T2DM Rats with ROCK inhibitor treatment

**Fig. 4:**
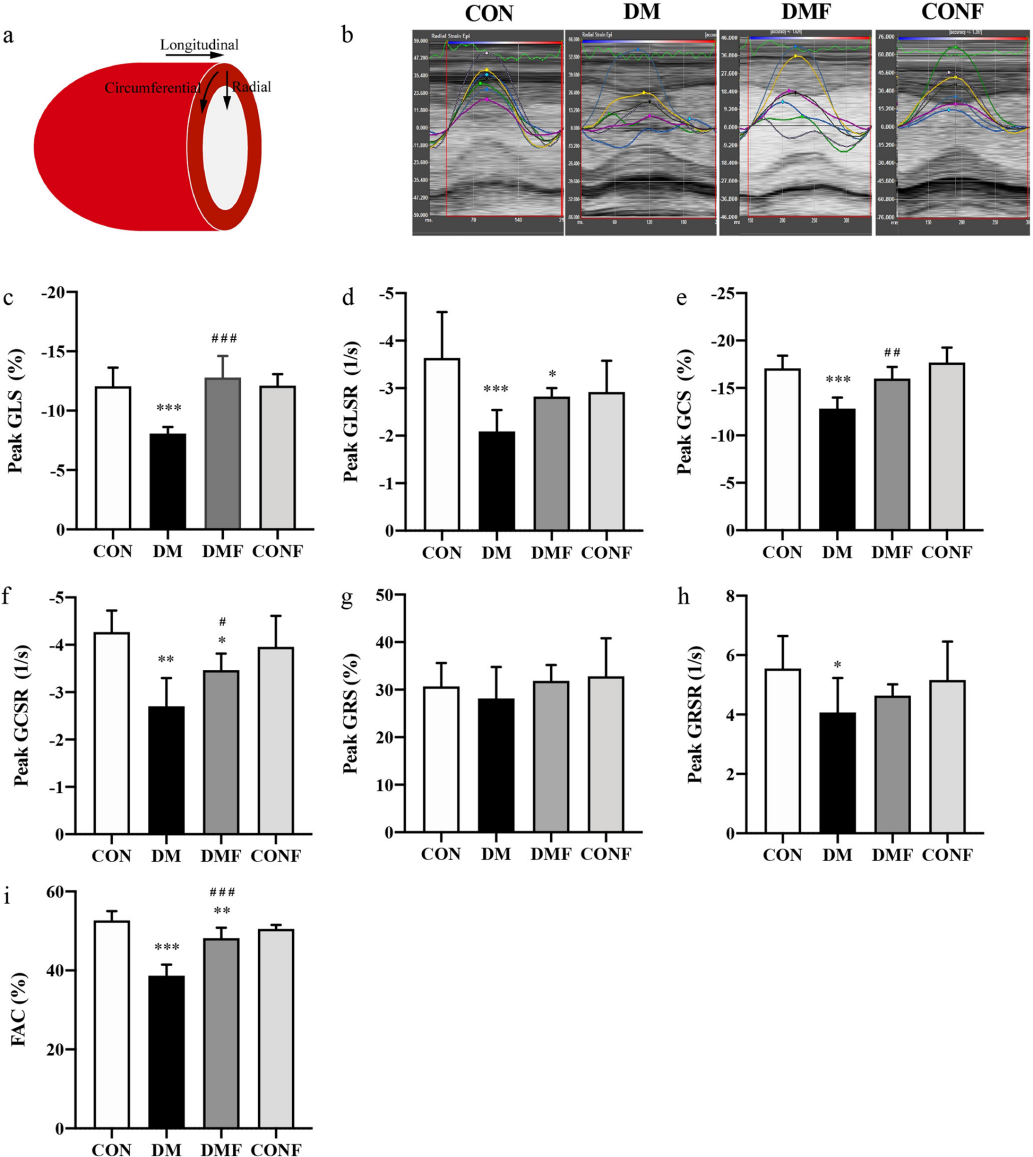
STE analysis in rat models. **a** A schematic of the LV with the indication of longitudinal, circumferential and radial strain measurements. **b** Representative longitudinal strain images of STE over one cardiac cycle. The peak values of **c** GLS, **d** GLSR, **e** GCS, **f** GCSR, **g** GRS, **h** GRSR and **i** FAC were calculated. GLS: global longitudinal strain, GLSR: global longitudinal strain rate, GCS: global circumferential strain, GCSR: global circumferential strain rate, GRS: global radial strain, GRSR: global radial strain rate, FAC: fractional area change. Values are presented as the means ± SDs. *P < 0.05, **P < 0.01, ***P < 0.001 *vs* CON group by one-way ANOVA; ^#^*P* < 0.05, ^##^*P* < 0.01, ^###^*P* < 0.001 *vs* DM group by one-way ANOVA

Myocardial strain parameters were measured by highly sensitive STE, which has been demonstrated to be a reliable approach to detect myocardial deformation and cardiac impairments (Fig. 4a, b and Table S3). Compared with the CON group, GLS and GLSR were markedly decreased in the T2DM rats, reflecting the damage of myocardial deformation in the longitudinal direction (*P* < 0.001, Fig. 4c and d). These impairments were prevented by fasudil, whereby GLS exhibited an approximately 58% increase but GLSR showed no significance (*P* < 0.001, Fig. 4c and d) in the DMF group. A similar pattern was found in the circumferential direction, where GCS and GCSR were reduced in the T2DM rats (*P* < 0.001, Fig. 4e and f), and both were significantly ameliorated in the DMF group (*P* < 0.05, Fig. 4e and f). In the radial direction, the strain rate revealed a significant change between the DM group and the CON group (*P* < 0.05, Fig. 4g and h), although the GRS and GRSR were both reduced. However, ROCK inhibition by fasudil had no significant effect on either GRS or GRSR in the DMF group (Fig. 4g and h). Additionally, FAC was attenuated in the T2DM rats (*P* < 0.001, Fig. 4i) and subsequently increased 24% in the DMF group (*P* < 0.001, Fig. 4i). Along with structural changes, cardiac function was slightly altered in ways that could be identified by myocardial strain parameters derived from STE.

### Diagnostic assessment of myocardial damage

**Fig. 5:**
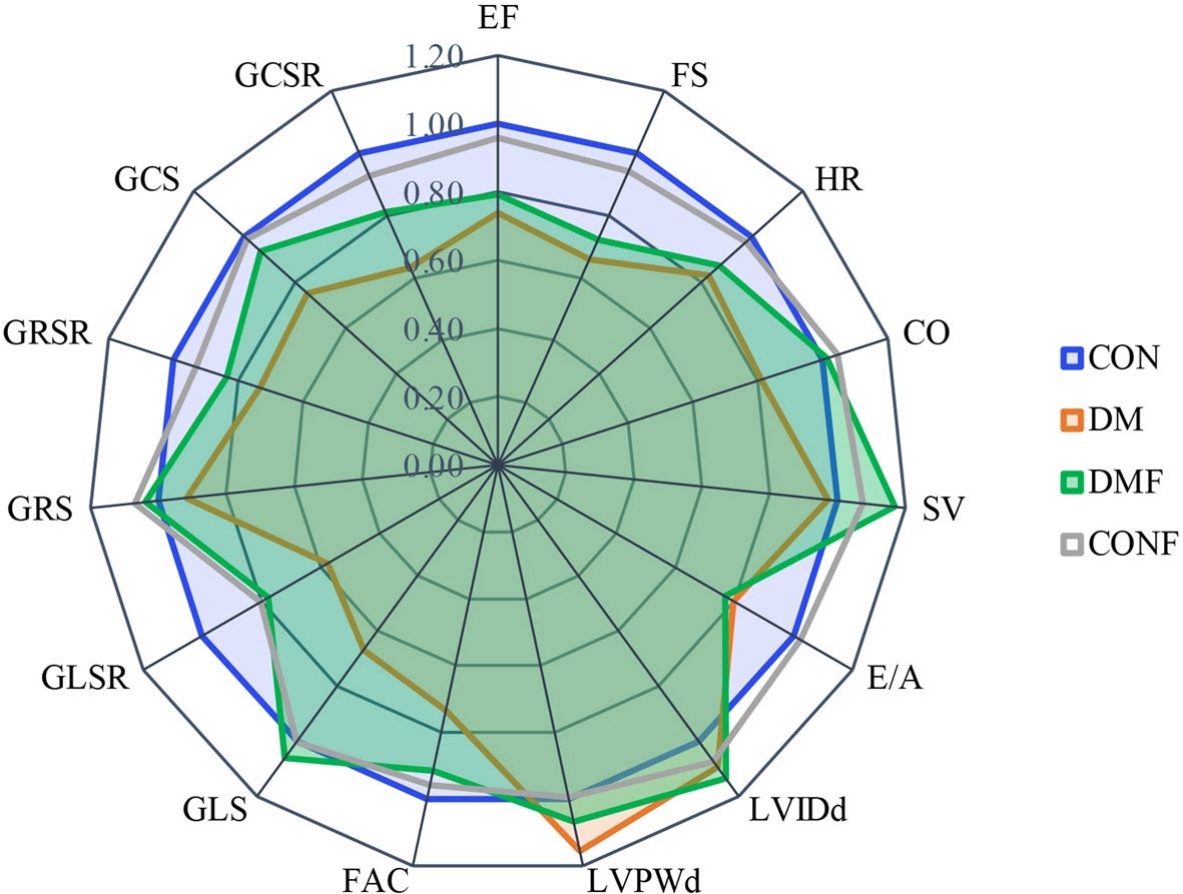
Cardiac function changes. The relative values of the treated and untreated diabetic rats in comparison to the healthy rats are shown. Healthy controls were normalized to one

Figure 5 summarizes the relative cardiac function alterations induced by diabetes and intervention compared with the control group by a spider’s web plot. Healthy controls are shown as a blue circle, which was normalized to 1. Changes in the DM group are marked by the red circle, and the DMF group is marked by the green circle. Similar deviations were observed in the treated and untreated T2DM rats. Notably, ROCK inhibition treatment in the DMF group tried to drive the green circle to the healthy shape by influencing several functional parameters and structural parameters (Fig. 5).

**Fig. 6:**
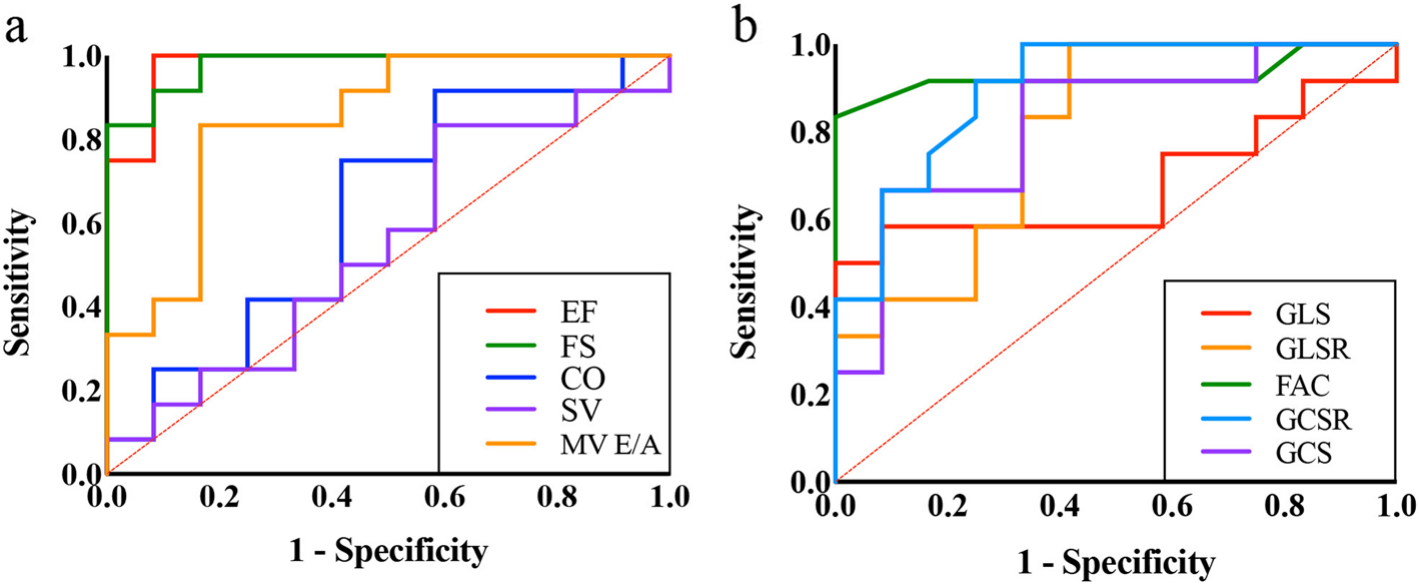
Receiver operating characteristic (ROC) curves for echocardiographic parameters to detect diabetic cardiomyopathy. **a** The ROC curves for conventional parameters and **b** STE parameters are shown. EF: ejection fraction; FS, fractional shortening, CO: cardiac output, SV: stroke volume, MV E/A: mitral valve E/A, GLS: global longitudinal strain, GLSR: global longitudinal strain rate, GCS: global circumferential strain, GCSR: global circumferential strain rate, FAC: fractional area change

To assess the validity of the parameters as predictors of diabetes-induced myocardial damage, ROC curves are displayed in Table 2. EF, FS and MV E/A had superior sensitivity and specificity for diabetic cardiomyopathy among the conventional parameters at cut-off values of 65.62%, 35.07% and 1.25, respectively (Fig. 6a and Table 2). Among STE parameters, FAC, GCS and GCSR were the best predictors of myocardial damage, with optimal cut-off values of 48%, -17.14% and -4.03 1/s, respectively (Fig. 6b and Table 2). GLSR and GRSR were also used to differentiate the cardiomyocytes with and without diabetes-induced damage (Table 2). Other STE parameters, GLS and GRS, failed to distinguish myocardial damage due to the lack of significance in the ROC curve analysis. Notably, the ability to predict myocardial damage was comparable between GLS and GLSR, which both indicate cardiac diastolic function (Table 3). The parameters characterizing cardiac systolic function, including FAC, GCS and GCSR, demonstrated nearly equivalent validity according to our study (Table 3). Furthermore, the predictive abilities of FAC and GCS were superior to that of GRS (*P* < 0.05, Table 3). These results indicate that FAC, GCS and GCSR were the sensitive and specific indices for detecting subtle changes in systolic dysfunction in the early stage of intervention.

**Table 2:** Receiver operating characteristics

Parameter	AUC (95% CI)	z statistic	*P* value	Optimal cut off	Sensitivity (95% CI)	Specificity (95% CI)
**EF**	0.979 (0.821 to 1.00)	20.39	< 0.001^***^	65.62(%)	100.00 (73.5—100.0)	91.67 (61.5—99.8)
**FS**	0.979 (0.821 to 1.00)	22.66	< 0.001^***^	35.07(%)	83.33 (51.6—97.9)	100.00 (73.5—100.0)
**CO**	0.639 (0.419 to 0.823)	1.17	0.243	89.75(ml/min)	75.00 (42.8—94.5)	58.33 27.7—84.8
**SV**	0.549 (0.334 to 0.750)	0.39	0.695	247.47(μl)	83.33 (51.6—97.9)	41.67 (15.2—72.3)
**MV E/A**	0.847 (0.643 to 0.960)	4.24	< 0.001^***^	1.25	83.33 (51.6—97.9)	83.33 (51.6—97.9)
**FAC**	0.927 (0.744 to 0.993)	6.40	< 0.001^***^	48(%)	83.33 (51.6—97.9)	100.00 (73.5—100.0)
**GLS**	0.681 (0.461 to 0.854)	1.48	0.138	-10.83(%)	58.33 (27.7—84.8)	91.67 (61.5—99.8)
**GLSR**	0.799 (0.586 to 0.933)	3.18	0.002^**^	-3.22(1/s)	100.00 73.5—100.0	58.33 (27.7—84.8)
**GCS**	0.819 (0.610 to 0.945)	3.53	< 0.001^***^	-17.14(%)	91.67 (61.5—99.8)	66.67 (34.9—90.1)
**GCSR**	0.899 (0.707 to 0.984)	6.34	< 0.001^***^	-4.03(1/s)	100.00 (73.5—100.0)	66.67 34.9—90.1
**GRS**	0.528 (0.316 to 0.733)	0.22	0.828	27.66(%)	33.33 (9.9—65.1)	91.67 (61.5—99.8)
**GRSR**	0.740 (0.522 to 0.895)	2.30	0.022^*^	4.93(1/s)	83.33 (51.6—97.9)	58.33 (27.7—84.8)

*AUC* area under the ROC curve, *CI* confidence interval, *EF* ejection fraction, *FS* fractional shortening, *CO* cardiac output, *SV* stroke volume, *MV E/A* mitral valve E/A, *FAC* fractional area change, *GLS* global longitudinal strain, *GLSR* global longitudinal strain rate, *GCS* global circumferential strain, *GCSR* global circumferential strain rate, *GRS* global radial strain, *GRSR* global radial strain rate. ROC analysis was applied to conventional parameters and STE parameters, respectively

^*^P < 0.05

^**^P < 0.01

^***^P < 0.001 *vs* AUC of 0.5

**Table 3:** Comparisons of the ROC curves

Variable	Difference between area	Standard Error	95% CI	z statistic	*P* value
**GLS vs GLSR**	0.118	0.117	-0.112 to 0.348	1.006	0.315
**GCS vs GCSR**	0.080	0.069	-0.055 to 0.214	1.164	0.244
**GRS vs GRSR**	0.212	0.101	0.014 to 0.410	2.099	0.036^*^
**GCS vs GRS**	0.292	0.138	0.020 to 0.563	2.108	0.035^*^
**GCSR vs GRSR**	0.160	0.104	-0.044 to 0.363	1.541	0.123
**FAC vs GCS**	0.108	0.073	-0.035 to 0.250	1.484	0.138
**FAC vs GCSR**	0.028	0.078	-0.126 to 0.181	0.354	0.723
**FAC vs GRS**	0.399	0.122	0.161 to 0.638	3.284	0.001^**^
**FAC vs GRSR**	0.188	0.110	-0.028 to 0.403	1.708	0.088

*FAC* fractional area change, *GLS* global longitudinal strain, *GLSR* global longitudinal strain rate, *GCS* global circumferential strain, *GCSR* global circumferential strain rate, *GRS* global radial strain, *GRSR* global radial strain rate. The comparison between the AUC of 2 ROC curves was performed. Statistically different

^*^P < 0.05

^**^P < 0.01

**Fig. 7:**
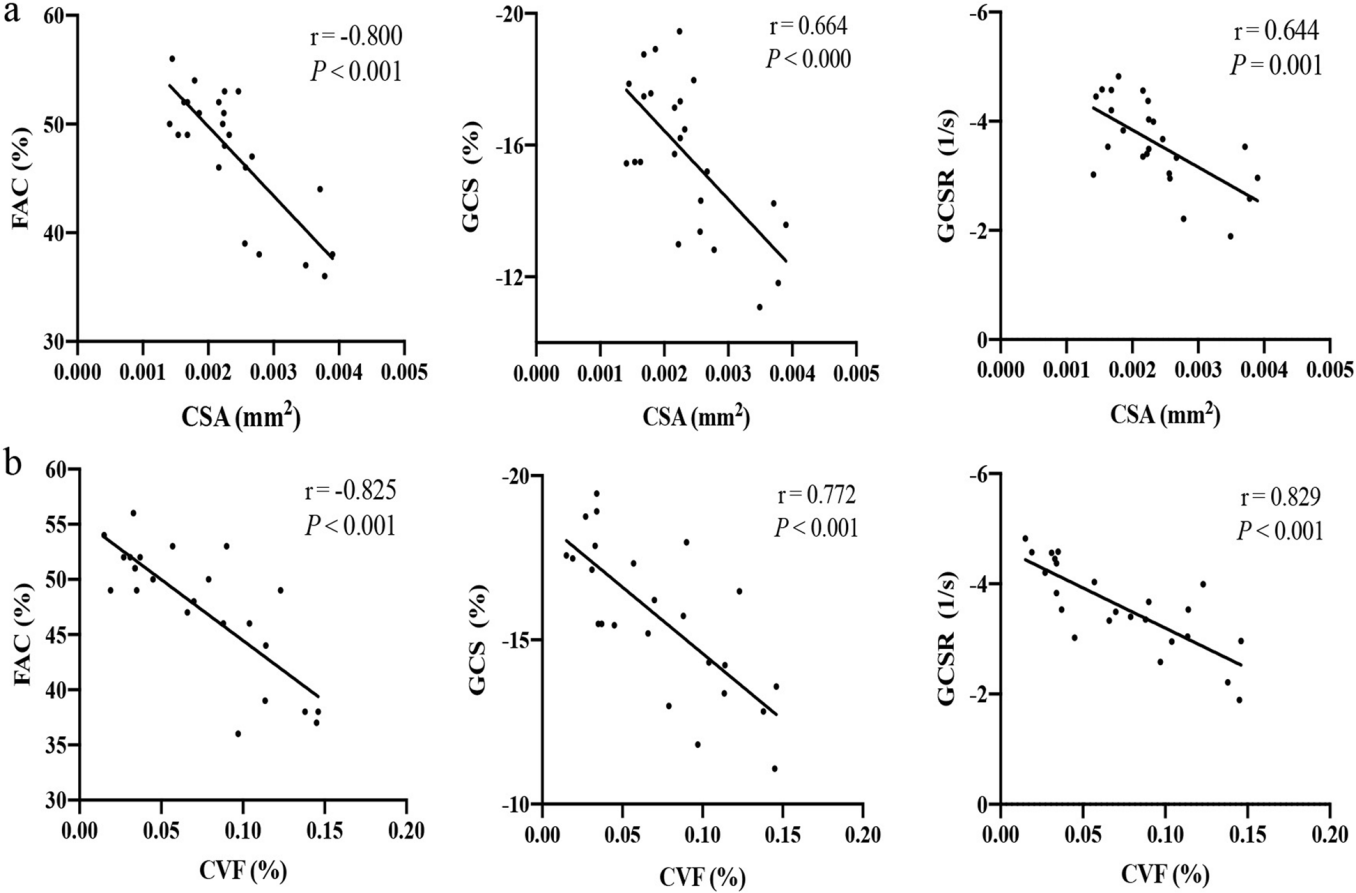
Correlation analysis. **a** The correlations between functional parameters and the CSA of cardiomyocytes. **b** The correlations between functional parameters and cardiac fibrosis. FAC: fractional area change, GCS: global circumferential strain, GCSR: global circumferential strain rate, CSA: cross sectional area, CVF: collagen volume fraction. r: linear regression coefficient. r and P values are shown for each linear regression

Linear regression analysis was performed to clarify the associations between cardiac function parameters and structure parameters (Table S4). FAC were strongly associated with diabetes-induced cardiac hypertrophy and fibrosis (Fig. 7a, b and Table S4). GCS and GCSR were intensively related to cardiac fibrosis but moderately related to cardiomyocyte hypertrophy (Fig. 7a, b and Table S4). The conventional parameters (EF and FS) and the STE parameters (GLS, GLSR and GRSR) all showed moderate correlations with cardiac hypertrophy and fibrosis, respectively (Table S4). In addition, CO and MV E/A showed low correlations with both cardiac structural parameters, and SV and GRS showed no significant relationships according to the analysis (Table S4). Among the functional parameters, FAC, GCS and GCSR were all useful for predicting cardiac hypertrophy and fibrosis, with FAC performing best.

## Discussion

The major findings in our study were that ROCK inhibition by fasudil improved diabetes-induced myocardial damage in both microstructure and mitochondrial dynamics. Accompanied by structural changes, cardiac function was ameliorated in T2DM rats treated with fasudil, and subtle changes could be identified by STE parameters, not conventional functional parameters. Myocardial strain and strain rate were more sensitive to early and subtle changes in cardiac function. According to the analysis, STE parameters were demonstrated to be potential predictors for cardiac hypertrophy and fibrosis, as well as preferable methods for detecting subtle alterations in cardiac dysfunction noninvasively and evaluating the therapeutic effect in the early stage.

Diabetic cardiomyopathy causes severe myocardial damage resulting in abnormalities in cardiac function and structure. Experimental and clinical studies have shown that cardiac hypertrophy and fibrosis both contribute to LV remodelling in diabetic animal models [2, 28]. In line with previous studies, we found significant augmentation of myocardial hypertrophy and fibrosis in T2DM rat hearts, which indicated that STZ-induced T2DM rats in our study developed cardiac remodelling under the long-term influence of the diabetic state.

Numerous molecular signalling pathways are involved in the pathophysiological process of diabetic cardiomyopathy [2–4, 28]. Recent studies have revealed the significant role of the RhoA/ROCK signalling pathway in the underlying mechanisms of diabetic cardiomyopathy [5, 6, 29]. Notably, Ocaranza et al. showed that T2DM patients treated with glucose-lowering drugs, antihypertensive treatment and statins had a significant increase in ROCK activation in peripheral blood along with increased plasma angiotensin II and malondialdehyde (MDA) levels, and inhibiting the rock cascade might induce clinical benefits in heart failure with reduced ejection fraction (HFrEF) patients. These results highlight the importance of the RhoA/ROCK signalling pathway in the prevention of T2DM. In the present study, we used fasudil, a ROCK inhibitor [30], to inhibit the RhoA/ROCK signalling pathway in T2DM rats. After 4 weeks of treatment, fasudil prominently alleviated cardiac hypertrophy and fibrosis, suggesting cardioprotective effects against cardiac remodelling.

Mitochondria provide energy for cardiomyocytes and are the power source of cardiac function [2, 4, 31]. Previous studies have proven that the RhoA/Rho kinase pathway is involved in the regulation of mitochondrial dynamics in some cases [29, 32–35]. To confirm the cardioprotective effect of fasudil on mitochondrial dynamics, the cardiac ultrastructure was confirmed by TEM. According to our experimental results, T2DM rat cardiomyocytes were characterized by dumbbell-shaped mitochondria for disturbed fusion, smaller fragments and even distorted vacuous mitochondria for exacerbated fission*.* ROCK inhibition by fasudil improved the disturbed mitochondrial fission/fusion in T2DM rats and facilitated mitochondrial fusion and uncoupling by upregulating Mfn1 and Mfn2 and downregulating Drp1 and Fis1. These results indicated that inhibiting the RhoA/ROCK signalling pathway could restore mitochondrial function and improve energy metabolism disorder in T2DM rat hearts, which contributed to systole and relaxation of the heart.

Correspondingly, we found impaired LV systolic and diastolic performance in T2DM rats. Our rat models shared numerous features with diabetic cardiomyopathy, including enlarged LV, remarkable myocardial hypertrophy and fibrosis and obvious dysfunction. Interestingly, ROCK inhibition by fasudil increased the SV and CO to a certain degree, possibly relevant to an integrated consequence of the slightly elevated LVEDV and HR. However, the classical parameters, EF, FS and MV E/A, derived from conventional echocardiography showed almost no change in the presence of 4 weeks of treatment with fasudil. The conventional parameters might be insensitive to subtle alterations in function along with improvements in structure in T2DM rats.

Because the results in our study and others have demonstrated the limitations of EF, FS and MV E/A in evaluating the early and subtle alterations of cardiac dysfunction [20, 36], we used STE to characterize diabetes-induced myocardial damage and assess the therapeutic effect in the early stage. Myocardial deformation measured by STE is broadly governed by myocardial microstructure. The LV longitudinal mechanics are predominantly determined by the subendocardium, while the circumferential and radial mechanics are determined by the mid-wall and the subepicardium [37, 38]. Myocardial strain is well known to account for ventricular vascular coupling, as decreased myocardial strain is known to attenuate myocardial efficiency [39]. Damage in different myocardial layers leads to distinct phenotypes in cardiac dysfunction [37, 40, 41], which makes it possible to classify heart disease according to layer-specific alterations. Injuries that occur mainly under the endocardium in animals and humans may be accompanied by diastolic dysfunction with a decrease in longitudinal mechanics, but radial and circumferential mechanics remain unchanged [17, 42]. Thus, GLS is a well-accepted marker of subendocardial damage and is linked to subendocardial fibrosis [43]. In addition, an acute transmural insult involving subepicardial and midmyocardial dysfunction results in a reduction in LV circumferential, radial and twist mechanics as well as a decrease in EF [37]. Consistent with this theory, Niu [16] found reduced longitudinal and radial mechanics in pressure overload-induced adult rats along with severely impaired contractile function, which was characterized by LV stiffness in all layers [16]. In diabetic humans and animal models, LV function was obviously impaired with reduced myocardial strain and strain rate [20, 44]. This was in accordance with our findings, where global strain and strain rate were markedly decreased in the T2DM rats in the longitudinal, circumferential and radial directions, suggesting transmural cardiac remodelling. Inhibiting the RhoA/ROCK signalling pathway significantly ameliorated GLS, GCS and GCSR. This finding suggested improvements in both cardiac diastolic and systolic function, as well as improvements in the myocardial microstructure in the global heart. FAC is well known in evaluating cardiac systolic function, reflecting the degree of thickening in the radial direction [37]. ﻿Reduced LV GLS and LV FAC suggested LV dysfunction and cardiac remodelling [15]. In our study, FAC was attenuated in T2DM rats and subsequently increased by ROCK inhibition treatment. Thus, fasudil ameliorated diabetes-induced cardiac systolic dysfunction in T2DM rats. Additionally, mitochondria are the powerhouse of the cell, which continuously provides large amounts of adenosine triphosphate (ATP) to cardiomyocytes to maintain cardiac function [31]. The improvements in mitochondrial function in energy metabolism also contributed to restoring the global strain and strain rate in the DMF group. Accordingly, it might be suggested that subtle changes in cardiac function could be detected by STE parameters at the early stage of treatment compared with conventional parameters. STE parameters could be suitable markers for predicting therapeutic effects and providing a quantitative assessment of layer-specific cardiac function alterations.

The present study demonstrated that fasudil improved diabetes-induced myocardial hypertrophy and fibrosis, and the amelioration of cardiac dysfunction accompanied by structural changes was detected by STE parameters (Fig. 6). However, which parameter is the optimal diagnostic approach to assess subtle cardiac damage and predict the therapeutic effect remains to be revealed. Hence, we demonstrated the validity of parameters as predictors for diabetic cardiomyopathy by ROC curves and linear regression analyses. Numerous studies have identified the sensitivity and specificity of conventional parameters for cardiovascular disease diagnosis [3, 45, 46]. Similar findings were confirmed in our study. However, the mild ability to indicate diabetic myocardial hypertrophy and fibrosis made conventional parameters fail to detect subtle alterations in cardiac function in the early stage of intervention. STE parameters were strongly associated with diabetes-induced cardiac hypertrophy and fibrosis than conventional parameters. That enabled subtle changes to be detected earlier by STE. According to our analysis, FAC, GCS and GCSR were sensitive and specific for cardiac structure changes and early and subtle alterations in cardiac function. Previous literature has shown that GLS parameters are detectable in corresponding microstructural and functional changes in the subendocardium and are associated with subendocardial fibrosis [17]. However, in our study, GLS and GLSR exhibited only a moderate relationship with cardiac fibrosis and hypertrophy in the whole fibre layers, which made it less significant for differentiating damaged and nondamaged cardiomyocytes in comparison with the other STE parameters. Nevertheless, GLS was obviously enhanced in the DMF group, suggesting probable improvements in cardiac diastolic function in the subendocardium. We further compared the area under the ROC curve for distinguishing the most suitable diagnostic markers for diabetic cardiomyopathy. Interestingly, FAC, GCS and GCSR were more specific and sensitive than the other parameters, and all three of them shared an equal capacity for the diagnosis of diabetes-induced cardiac systolic dysfunction. Therefore, FAC, GCS and GCSR were more sensitive and specific for subtle changes in systolic dysfunction in the early stage of intervention. STE parameters were demonstrated to be potential predictors for the detection of diabetes-induced myocardial damage. Early detection is feasible and might be predictive of the therapeutic effect in diabetic cardiomyopathy.

## Limitations

Several possible limitations should be considered. First, the drug for diabetes treatment is a major limitation. Although the RhoA/ROCK signalling pathway is of increasing importance in cardiovascular disease therapy, ROCK inhibition is not the classic method for diabetic cardiomyopathy therapy compared with glucose control. We will improve animal models and experimental design in follow-up studies, for providing more preclinical data. Second, the current number of investigated rats is low, which serves as a limitation. Although the sample size is based on statistical calculations according to previous observations, there may still increase the risk of error in the experimental results. The sample size should be expanded to solve this problem in future research. Finally, although we discussed the ability of STE parameters to indicate damage in different fibre layers, we did not correlate the functional parameters with structural parameters in different myocardial fibre layers. Further research needs to consider the matter.

## Conclusion

In conclusion, our data suggested that short-term ROCK inhibition improved diabetes-induced myocardial damage in both the microstructure and mitochondrial dynamics in T2DM rats. STE parameters were demonstrated to be potential predictors for cardiac hypertrophy and fibrosis compared with conventional parameters. FAC, GCS and GCSR are more sensitive and specific, which makes them preferable for the early detection and quantification of subtle and layer-specific functional alterations. Our study provides translational evidence for a better understanding of the pathophysiologic progress of diabetic cardiomyopathy during management.

## Supplementary Information

**Additional file 1.**

## Data Availability

All data generated or analyzed during this study are included in this published article.

## Abbreviations

DM: Diabetes mellitus
T2DM: Type 2 diabetes mellitus
HF: Heart failure
HFrEF: Heart failure with reduced ejection fraction
STE: Speckle-tracking echocardiography
HFD: High-fat diet
STZ: Streptozocin
TEM: Transmission electron microscopy
ROC: Receiver operating characteristic
BW: Body weight; FBG: fasting blood glucose
HR: Heart rate; HW: heart weight
HW/BW: Heart weight / body weight
HW/TL: Heart weight / tibia length
CSA: Cross sectional area
CVF: Collagen volume fraction
EF: Ejection fraction
FS: Fractional shortening
CO: Cardiac output
SV: Stroke volume
MV E/A: Mitral valve E/A
LVPWd: Diastolic left ventricular posterior wall thickness
LVPWs: Systolic left ventricular posterior wall thickness
LVIDd: Left ventricular end-diastolic inner dimension
LVIDs: Left ventricular end-systolic inner dimension
LVEDV: Left ventricular end-diastolic volume
LVESV: Left ventricular end-systolic volume
GLS: Global longitudinal strain
GLSR: Global longitudinal strain rate
GCS: Global circumferential strain
GCSR: Global circumferential strain rate
FAC: Fractional area change
GRS: Global radial strain
GRSR: Global radial strain rate
ROCKs: Rho-associated coiled-coil containing kinases
