# Supplementary material for: Speckle-tracking echocardiography provides sensitive measurements of subtle early alterations associated with cardiac dysfunction in T2DM rats
Source: BMC Cardiovasc Disord. 2023 May 22;23:266. doi: 10.1186/s12872-023-03239-2 (PMC10201756; doi:10.1186/s12872-023-03239-2)
Supplement: Supplementary file 1 — Additional file 1. [file 12872_2023_3239_MOESM1_ESM.pdf]

# Speckle-Tracking Echocardiography Provides Sensitive Measurements of Subtle Early Alterations Associated with Cardiac Dysfunction in T2DM Rats

## Supplementary Materials

## Methods

### Quantitative real time PCR (qRT-PCR)

Total mRNA of the samples from animal hearts were extracted using TRIzol reagent (Invitrogen, Carlsbad, CA) according to the manufacturer's protocol[1]. QRT-PCR was performed by using SYBR Green to detect PCR products in real time with ABI PRISM 7700 Sequence Detection System (Applied Biosystems). The relative expression levels of the genes were calculated by the $2^{-\Delta\Delta CT}$ method and β-actin served as a housekeeping gene. The sequences of the primers were used as follow:

Table S1 The sequences of the primers used in this study.

| Gene (rat) | Forward primer (5'->3') | Reverse primer (5'->3') |
|---|---|---|
| **Mfn1** | GCTGCATACAGACAGACAGCCT | GGTAATGACCTGTCTCAGGGCT |
| **Mfn2** | CACTACCACATCGGACACCCTA | GAACTTGTGTCTTGCATTTGGC |
| **Drp1** | AGGAACCGACAACAGGCAACT | CAACTGGAACTGGCACATCTAGC |
| **Fis1** | TACCCCGAGGCTGTCCTAAG | CAGGACATTAGGCCCAGAGC |
| **β-actin** | GCAGGAGTACGATGAGTCCG | ACGCAGCTCAGTAACAGTCC |

1. Yu J, He J, Yang W, Wang X, Shi G, Duan Y, Wang H, Han C. Diabetes impairs the protective effects of sevoflurane postconditioning in the myocardium subjected to ischemia/ reperfusion injury in rats: important role of Drp1. BMC Cardiovasc Disord. 2021;21(1):96.

**Figures:**

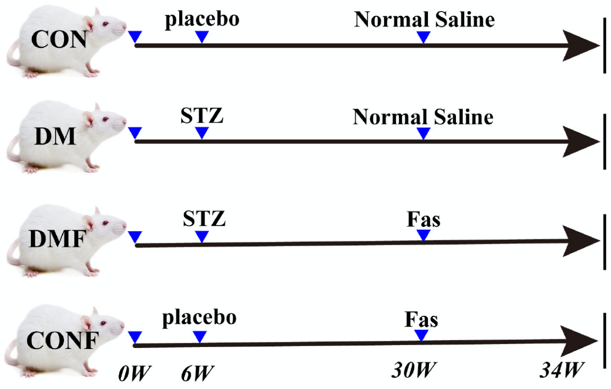


Fig.S1 A schematic of the experimental protocol.

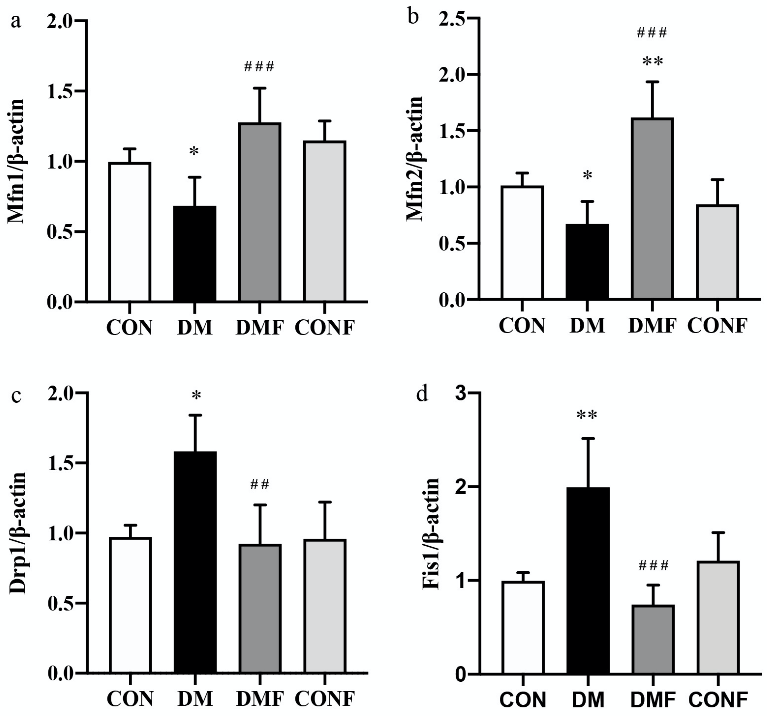


Fig.S2 Effect of ROCK inhibition by fasudil on mitochondrial fusion and fission. The expression levels of the genes (a) Mfn1 and (b) Mfn2 were reduced in the DM group but increased in the DMF group. The transcripts of the genes (c) Drp1 and (d) Fis1 were upregulated in diabetic rats but reduced after treated with fasudil. Values are presented as the means ± SDs. *$P < 0.05$, **$P < 0.01$, ***$P < 0.001$ *vs* CON group by one-way ANOVA; $^{\#}P < 0.05$, $^{\#\#}P < 0.001$, $^{\#\#\#}P < 0.001$ *vs* DM group by one-way ANOVA.

**Tables:**

Table S2. Conventional echocardiographic parameters of cardiac function.

| | CON | DM | DMF | CONF |
|---|---|---|---|---|
| **HR (bmp)** | 354 ± 29 | 295 ± 48** | 309 ± 20* | 346 ± 25 |
| **LVPWd (mm)** | 1.82 ± 0.07 | 2.11 ± 0.15** | 1.95 ± 0.19 | 1.81 ± 0.19 |
| **LVPWs (mm)** | 3.34 ± 0.37 | 2.90 ± 0.19* | 3.07 ± 0.24 | 3.09 ± 0.28 |
| **LVIDd (mm)** | 8.01 ± 0.46 | 8.76 ± 0.75* | 9.09 ± 0.76** | 8.60 ± 0.40 |
| **LVIDs (mm)** | 4.30 ± 0.41 | 6.11 ± 0.69*** | 6.07 ± 0.67*** | 4.87 ± 0.65 |
| **LVEDV (μl)** | 347 ± 45 | 425 ± 81 | 461 ± 90** | 405 ± 41 |
| **LVESV (μl)** | 84 ± 18 | 190 ± 49*** | 187 ± 49*** | 114 ± 34 |
| **EF (%)** | 75.39 ± 6.64 | 55.68 ± 3.95*** | 59.75 ± 4.33*** | 72.27 ± 6.52 |
| **FS (%)** | 46.18 ± 6.50 | 30.41 ± 2.67*** | 33.39 ± 3.06*** | 43.45 ± 5.70 |
| **CO (ml/min)** | 94.61 ± 14.74 | 76.67 ± 15.64* | 95.90 ± 11.49# | 99.00 ± 16.21 |
| **SV (μl)** | 267 ± 31 | 259 ± 34 | 312 ± 46 # | 287 ± 47 |
| **MV E/A** | 1.53 ± 0.23 | 1.22 ± 0.10* | 1.18 ± 0.24* | 1.57 ± 0.35 |

HR: heart rate, LVPWd: diastolic left ventricular posterior wall thickness, LVPWs: systolic left ventricular posterior wall thickness, LVIDd: left ventricular end-diastolic inner dimension, LVIDs: left ventricular end-systolic inner dimension, LVEDV: Left ventricular end-diastolic volume, LVESV: left ventricular end-systolic volume, EF: ejection fraction, FS: fractional shortening, CO: cardiac output, SV: stroke volume, MV E/A: mitral valve E/A. Values are presented as the means ± SDs. *$P < 0.05$, **$P < 0.01$, ***$P < 0.001$ *vs* CON group by one-way ANOVA; #$P < 0.05$, ##$P < 0.001$, ###$P < 0.001$ *vs* DM group by one-way ANOVA.

Table S3. Speckle tracking echocardiography characteristics of the LV.

| | | CON | DM | DMF | CONF |
|---|---|---|---|---|---|
| Longitudinal | V PK(cm/s) | 1.03±0.47 | 0.72±0.26 | 0.85±0.34 | 0.85±0.32 |
| | D PK(cm/s) | 0.16±0.06 | 0.20±0.09 | 0.29±0.12$^{*}$ | 0.25±0.11 |
| | GLS PK(%) | -12.06±1.57 | -8.07±0.55$^{***}$ | -12.79±1.81$^{\#\#\#}$ | -12.10±0.98 |
| | GLSR PK(1/s) | -3.63±0.97 | -2.09±0.45$^{***}$ | -2.82±0.18$^{*}$ | -2.92±0.66 |
| Circumferential | V PK(cm/s) | 101.60±50.13 | 62.64±26.23$^{*}$ | 70.62±26.00 | 69.52±12.09 |
| | D PK(cm/s) | 2.31±1.55 | 1.07±0.63 | 2.17±1.25 | 1.66±0.77 |
| | GCS PK(%) | -17.08±1.32 | -12.82±1.18$^{***}$ | -15.98±1.24$^{\#\#}$ | -16.90±2.38 |
| | GCSR PK(1/s) | -4.27±0.46 | -2.70±0.60$^{***}$ | -3.46±0.35$^{*\#}$ | -3.96±0.65 |
| Radial | As ($mm^2$) | 21.48±1.46 | 42.93±4.94$^{***}$ | 29.88±4.02$^{***\#\#\#}$ | 26.15±1.74$^{*}$ |
| | Ad ($mm^2$) | 45.95±2.57 | 70.37±6.04$^{***}$ | 58.18±7.19$^{**\#\#}$ | 53.40±3.15$^{*}$ |
| | FAC (%) | 52.67±2.34 | 38.67±2.80$^{***}$ | 48.17±2.64$^{**\#\#\#}$ | 50.50±1.05 |
| | V PK(cm/s) | 1.66±0.13 | 1.36±0.27$^{*}$ | 1.45±0.20 | 1.69±0.23 |
| | D PK(cm/s) | 0.75±0.06 | 0.69±0.06 | 0.77±0.09 | 0.81±0.10 |
| | GRS PK(%) | 30.69±4.91 | 28.14±6.65 | 31.87±3.30 | 32.82±7.98 |
| | GRSR PK(1/s) | 5.55±1.10 | 4.07±1.16$^{*}$ | 4.64±0.38 | 5.16±1.30 |

V: velocity, D: displacement, PK: peak value, GLS: global longitudinal strain, GLSR: global longitudinal strain rate, GCS: global circumferential strain, GCSR: global circumferential strain rate, As: area at systole, Ad: area at diastole, FAC: fractional area change, GRS: global radial strain, GRSR: global radial strain rate. Values are presented as the means ± SDs. $^{*}P < 0.05$, $^{**}P < 0.01$, $^{***}P < 0.001$ *vs* CON group by one-way ANOVA; $^{\#}P < 0.05$, $^{\#\#}P < 0.001$, $^{\#\#\#}P < 0.001$ *vs* DM group by one-way ANOVA.

Table S4. The correlations between cardiac functional parameters and cardiac structural parameters.

| | | EF | FS | CO | SV | E/A | FAC | GLS | GLSR | GCS | GCSR | GRS | GRSR |
|---|---|---|---|---|---|---|---|---|---|---|---|---|---|
| **CSA** | **Pearson Correlation** | -0.631 | -0.602 | -0.497 | -0.123 | -0.468 | -0.800 | 0.569 | 0.475 | 0.664 | 0.644 | -0.207 | -0.451 |
| | **Sig. (2-tailed)** | **0.001** | **0.002** | **0.014** | 0.566 | **0.021** | **<0.001** | **0.004** | **0.019** | **<0.001** | **0.001** | 0.331 | **0.027** |
| **CVF** | **Pearson Correlation** | -0.691 | -0.661 | -0.496 | -0.16 | -0.549 | -0.825 | 0.622 | 0.580 | 0.772 | 0.829 | -0.08 | -0.526 |
| | **Sig. (2-tailed)** | **<0.001** | **<0.001** | **0.014** | 0.457 | **0.005** | **<0.001** | **0.001** | **0.003** | **<0.001** | **<0.001** | 0.712 | **0.008** |

EF: ejection fraction; FS, fractional shortening, CO: cardiac output, SV: stroke volume, MV E/A: mitral valve E/A, FAC: fractional area change, GLS: global longitudinal strain, GLSR: global longitudinal strain rate, GCS: global circumferential strain, GCSR: global circumferential strain rate, GRS: global radial strain, GRSR: global radial strain rate, CSA: cross sectional area, CVF: collagen volume fraction.
